# Evaluating dopamine transporter imaging as an enrichment biomarker in a phase 2 Parkinson’s disease trial

**DOI:** 10.1186/s12883-021-02470-8

**Published:** 2021-11-23

**Authors:** R. Matthew Hutchison, Karleyton C. Evans, Tara Fox, Minhua Yang, Jerome Barakos, Barry J. Bedell, Jesse M. Cedarbaum, Miroslaw Brys, Andrew Siderowf, Anthony E. Lang

**Affiliations:** 1 Biogen, 300 Binney Street, Cambridge, MA 02142 USA; 2 Biogen, Maidenhead, UK; 3 Bioclinica, Princeton, NJ USA; 4 Biospective Inc., Montreal, QC Canada; 5 Coeruleus Clinical Sciences LLC, Woodbridge, CT USA; 6 Eli Lilly and Company, Indianapolis, IN USA; 7 University of Pennsylvania, Philadelphia, PA USA; 8 Morton and Gloria Shulman Movement Disorders Clinic, Toronto, ON Canada; 9 Edmond J. Safra Program in Parkinson’s Disease, Toronto, ON Canada

**Keywords:** Biomarker, Dopamine transporter, Parkinson’s disease, SPECT, SWEDD

## Abstract

**Background:**

Dopamine transporter single-photon emission computed tomography (DaT-SPECT) can quantify the functional integrity of the dopaminergic nerve terminals and has been suggested as an imaging modality to verify the clinical diagnosis of Parkinson’s disease (PD). Depending on the stage of progression, approximately 5–15% of participants clinically diagnosed with idiopathic PD have been observed in previous studies to have normal DaT-SPECT patterns. However, the utility of DaT-SPECT in enhancing early PD participant selection in a global, multicenter clinical trial of a potentially disease-modifying therapy is not well understood.

**Methods:**

The SPARK clinical trial was a phase 2 trial of cinpanemab, a monoclonal antibody against alpha-synuclein, in participants with early PD. DaT-SPECT was performed at screening to select participants with DaT-SPECT patterns consistent with degenerative parkinsonism. Acquisition was harmonised across 82 sites. Images were reconstructed and qualitatively read at a central laboratory by blinded neuroradiologists for inclusion prior to automated quantitative analysis.

**Results:**

In total, 482 unique participants were screened between January 2018 and May 2019; 3.8% (15/398) of imaged participants were excluded owing to negative DaT-SPECT findings (i.e., scans without evidence of dopaminergic deficit [SWEDD]).

**Conclusion:**

A smaller proportion of SPARK participants were excluded owing to SWEDD status upon DaT-SPECT screening than has been reported in prior studies. Further research is needed to understand the reasons for the low SWEDD rate in this study and whether these results are generalisable to future studies. If supported, the radiation risks, imaging costs, and operational burden of DaT-SPECT for enrichment may be mitigated by clinical assessment and other study design aspects.

**Trial registration:**

ClinicalTrials.gov identifier: NCT03318523.

Date submitted: October 19, 2017.

First Posted: October 24, 2017.

## Background

Interventional clinical trials of disease-modifying therapies for Parkinson’s disease (PD) have begun enrolling participants at earlier stages of disease to increase the probability of demonstrating drug efficacy. Because clinical diagnosis is less accurate at the onset of PD, leading to underdiagnosis or misdiagnosis, the consideration of biomarkers to identify participants most likely to benefit from intervention has become a critical aspect of trial design [1–4]. Dopamine transporter single-photon emission computed tomography (DaT-SPECT) is a promising imaging modality for clinical trial enrichment based on the progressive degeneration of dopaminergic neurons that characterises PD. The use of DaT-SPECT to verify a clinical diagnosis of PD has increased in recent years, both in clinical practice settings and in clinical trials [5–11].

Radiotracers for positron emission tomography (PET) and SPECT have been developed for molecular imaging of DaT protein density to allow for the in vivo visualisation and quantification of the functional integrity of the dopaminergic nerve terminals. In participants with early PD, striatal DaT signal loss of approximately 30–50% has been shown [12–14], and the density further decreases as participants progress to more severe stages of the disease [15, 16]. ^123^I-ioflupane (also known as ^123^I-FP-CIT or by its tradename DaTscan™ [GE Healthcare, Chicago, IL, USA]) is the most widely available and commonly used DaT-SPECT tracer. Prior work has suggested DaT-SPECT has a sensitivity range of 79–100% and specificity of 80–100% when using clinical diagnosis as the reference [17–19]. The value of DaT-SPECT can be appreciated when the added assessment of dopaminergic nerve terminal integrity serves to refine the differential diagnosis given the overlap of clinical manifestations associated with parkinsonism not caused by neurodegenerative nigrostriatal cell loss (e.g., functional parkinsonism, dystonic tremor, dopa-responsive dystonia, and drug-induced parkinsonism).

Previous studies that deployed DaT-SPECT during screening identified participants who met the trial-specific clinical criteria of PD but excluded a fraction of participants owing to negative DaT-SPECT findings (i.e., scans without evidence of dopaminergic deficit [SWEDDs]) [6–9]. Study inclusion criteria often include a neurologist’s assessment of the presence of cardinal motor symptoms of PD (e.g., United Kingdom Parkinson’s Disease Society Brain Bank clinical diagnostic criteria; Gibb and Lees, 1988) [20, 21] and meeting pre-specified thresholds on the Hoehn and Yahr scale or Unified Parkinson’s Disease Rating Scale (UPDRS). It has additionally been postulated that a portion of SWEDD participants might have conditions resembling degenerative parkinsonism but, in fact, present with medication-induced or vascular parkinsonism or other conditions, implying they have a lower probability of benefiting from dopaminergic therapy and show a lower probability of progressing in clinical motor disability [10, 19, 22, 23]. Participants with SWEDD are estimated to represent 5–15% of the population of participants in clinical trials and up to 20% of participants in observational cohorts [24, 25]. Of note, the SWEDD percentage varies by the average disease duration of recruited participants [10, 25]. Clinical trial simulations based on drug trial (PRECEPT, mean disease duration = ~ 8 months) [26] and natural history (Parkinson’s Progression Markers Initiative [PPMI], mean disease duration = ~ 7 months) [27] data of early PD participants suggest a 24% reduction of sample size could be achieved when trials are enriched by including only DaT-deficient participants and excluding the SWEDD participants [23]. Given the disease-specific decline of DaT-SPECT signal and ability to identify SWEDDs, DaT-SPECT has received regulatory approval from the US Food and Drug Administration and the European Medicines Agency (EMA) to aid in the differential diagnosis of PD [28, 29] and has been recently qualified as a clinical trial enrichment biomarker for PD trials by the EMA [30].

The EMA biomarker qualification and published reports provide converging evidence to support the use of DaT-SPECT as an enrichment biomarker. However, it is not trivial to implement DaT-SPECT as a screening biomarker in large clinical trials. Upon considering the value of deploying DaT-SPECT to confirm the diagnosis of PD in study participants, several other factors deserve consideration, including imposed burden to participants (time, radiation exposure), DaT-SPECT global scalability and standardisation, and increased operational cost and burden. Although DaT-SPECT can aid in the differentiation of PD participants from healthy controls and from other non-PD disorders, studies have shown the sensitivity and specificity of DaT-SPECT may not be sufficient to improve diagnostic accuracy over clinical assessment alone [31–33]. Only limited prior work has included neuropathologic validation to ascertain the true diagnosis of participants [34, 35], and the possible synergistic effects of combining both imaging and clinical assessments are often not evaluated. Moreover, the comparison of performance metrics for DaT-SPECT versus clinical diagnosis will vary by clinical setting [36]. In an effort to provide additional insight into the value of DaT-SPECT as an enrichment biomarker in early PD clinical trials, this report evaluated the incidence of SWEDDs in a large, multisite, interventional clinical trial.

## Material and methods

### Study overview

The SPARK study (NCT03318523) was a randomised, double-blind, placebo-controlled, parallel-group, phase 2 study entitled, ‘Evaluating the Efficacy, Safety, Pharmacokinetics, and Pharmacodynamics of BIIB054 in Participants With Parkinson’s Disease.’ BIIB054 (cinpanemab) is a monoclonal antibody that preferentially binds to aggregated forms of alpha-synuclein, a major constituent of Lewy bodies, which are thought to play a central role in the pathophysiology of PD and progression of the disease [37]. The study was designed to enrol untreated participants aged 40–80 years diagnosed with PD in the previous 3 years. Similar to the PPMI criteria, PD diagnosis for SPARK required asymmetric or bilateral presentation of either resting tremor and bradykinesia, bradykinesia and rigidity, or rigidity and resting tremor; or either asymmetric resting tremor or asymmetric bradykinesia. Further, SPARK inclusion criteria required participants to have a DaT-SPECT scan showing evidence of striatal dopaminergic deficit. Participant inclusion and exclusion criteria were explicitly outlined in the trial protocol to harmonise study participant recruitment across all clinical sites. The SPARK study was terminated because it failed to meet the primary and secondary outcome measures.

### Participants

Study enrollment began in January 2018 at 82 sites across nine countries, and participant randomisation was completed in May 2019; 495 participants were screened, of which 398 (from 77 sites) proceeded to DaT-SPECT, the final assessment in the screening sequence.

### Standard protocol approvals, registrations, and patient consents

The study was done in accordance with applicable International Conference on Harmonisation and Good Clinical Practice Guidelines. Ethics approval was granted by each centre’s local or national independent ethics committee. Written informed consent was obtained from all patients (or guardians of patients) participating in the study.

### Data availability statement

Data are available on request at http://clinicalresearch.biogen.com.

### DaT-SPECT

#### Image acquisition

In accordance with DaTscan™ guidelines, a minimum of 111 MBq (3 mCi) ^123^I-ioflupane was required for injection. The target dose was set to 185 MBq (5 mCi) to allow for optimal image quantification. If the injected dose was less than 166.5 MBq (4.5 mCi), the frame duration was increased to achieve sufficient photopeak counts (1.5 million). At least 1 h prior to the intravenous administration of ^123^I-ioflupane, thyroid blockade was performed to reduce the uptake of the ligand by the thyroid (pursuant to local regulation and practice). Following previous work [33, 38–40], participants were imaged within a 4-h (± 30 min) window following the ^123^I-ioflupane injection.

Across study sites, SPECT imaging procedures were prospectively harmonised to require fitting of gamma cameras with at least two high-resolution parallel-hole or fan-beam low-energy high-resolution or low-energy ultra–high-resolution collimators, one per head (photopeak of 159 keV, ± 10% energy window). Detector heads were oriented at 180 degrees, utilised a circular orbit within a 15-cm radius in clockwise step-and-shoot mode, and sampled ≥120 angular views over 360° (~ 34 min). Prior to participant enrollment, each imaging site underwent a qualification procedure that included camera sensitivity assessment via a striatal phantom and acquisition optimisation via adjustment of several parameters (i.e., zoom, matrix size, and step duration).

Raw projection data across all sites was exported to a standalone Hermes workstation (Hermes GOLD, workstation release 1.4, Hybrid Recon-Neurology package version 1.3), after which motion correction, ordered subset expectation maximisation iterative reconstruction (iterations and subsets = 10i10s), and attenuation correction (uniform correction via the Chang 0 method; μ = 0.11 cm^− 1^) [41] were performed. Similar to other large multicenter studies, no resolution recovery, scatter correction, or filtering was applied during imaging processing [30].

#### Visual reads

Following reconstruction, each DaT-SPECT image was assessed for dopamine transporter deficit (in accordance with the DaTscan™ label) via independent visual read by two neuroradiologists based at the central lab. The readers were board-certified in radiology and granted certification of added qualification in the subspecialty of neuroradiology from the American Board of Radiology. Readers were active in clinical practice and clinical research. A DaT deficit was identified when the results demonstrated that activity in the striatum was either asymmetric, absent in the putamen and/or one or both caudate nuclei, and consistent with neurodegenerative parkinsonism. Each reader was blinded to the results of the other. However, if the two reads for a given scan were not in agreement (either “normal” or “abnormal”), the readers then performed an unblinded consultation to reach a consensus read. Readers were aware that the reads were being performed as part of a clinical drug study.

#### Striatal binding ratio quantification

Image quantification was performed using a proprietary, configurable, modular, pipeline-based system that allows for fully automated high-throughput processing of multi-modality images (PIANO™; Biospective Inc., Montreal, QC, Canada; https://biospective.com/imaging-core-lab). DaT-SPECT images were registered to each participant’s own anatomical three-dimensional T1-weighted magnetic resonance imaging (3D-T1 MRI) scan using a participant- and visit-specific pseudo–DaT-SPECT scan. The pseudo–DaT-SPECT scan was generated from the 3D-T1 MRI scan using a process that scales the caudate/putamen voxels of the 3D-T1 MRI volume and smooths the scaled image to be similar to an actual DaT-SPECT image. Following linear registration (using a cost function based on mutual information) of the SPECT to the pseudo–DaT-SPECT image, a nonlinear template-to-DaT-SPECT transformation was generated by combining the linear T1-to-DaT-SPECT and nonlinear template-to-T1 transformations derived from the 3D-T1 anatomical MRI registration. Regions of interest (ROI) were obtained from an anatomical atlas defined on the study-specific template. The ROIs were mapped onto the DaT-SPECT image and used to collect ROI-wise striatal binding ratio (SBR) values using the occipital lobe as the reference region, calculated as (ROI value / occipital reference value) – 1.

Putamen and caudate SBR values were demographically indexed by dividing the SBR by values that have been published for healthy volunteers based on age and sex [42, 43]. For the putamen, this was calculated as SBR / (6.702–0.0339 × age) for males and SBR / (7.116–0.0339 × age) for females. For caudate, this was calculated as SBR / (6.8–0.0273 × age) for males and SBR / (7.232–0.0273 × age) for females. Age-dependent striatum, caudate nucleus, and putamen uptake values and the striatal asymmetry index were also calculated. Following previous work, abnormal uptake was considered when below 3.93074–0.02156 × age for the striatum, 3.79744–0.02168 × age for the putamen, and 4.03099–0.02141 × age for the caudate nucleus; a striatal asymmetry index > 12.22 was considered abnormal [33, 38–40].

## Results

### Study population characteristics

Available demographics and clinical scores for the screened participants who did and did not undergo a DaT-SPECT scan are shown in Table 1. Of the screened participants that were excluded prior to completion of the DaT-SPECT scan, the most common screen fails were related to a low estimated glomerular filtration rate, the screening assessment process exceeding the allowed screening window, a Montreal Cognitive Assessment score < 23, MRI abnormalities, and participants being currently on excluded medications.

**Table 1:** Baseline demographics and clinical characteristics

	Screen failed prior to DaT-SPECT collection	DaT-SPECT screened	DaT-SPECT “abnormal”	DaT-SPECT “normal”
*N*	85	398	383	15
Male	54 (63.5)	280 (70.5)	269 (70.4)	11 (73.3)
Caucasian	75 (88.2)	360 (90.7)	348 (91.1)	12 (80.0)
Age at enrollment, y	63.8 ± 9.73	60.0 ± 8.99	60.1 ± 9.00	58.6 ± 8.96
PD classification				
TD	53 (67.1)	281 (70.8)	273 (71.5)	8 (53.3)
PIGD	22 (27.8)	84 (21.2)	79 (20.7)	5 (33.3)
Indeterminate	4 (5.1)	32 (8.1)	30 (7.9)	2 (13.3)
Time since disease onset at enrollment, y	2.5 ± 2.34	1.9 ± 1.76	1.9 ± 1.72	2.7 ± 2.69
Time since PD diagnosis, y	0.6 ± 0.62	0.7 ± 0.64	0.7 ± 0.64	0.5 ± 0.59
Prior PD medication history^a^	9 (10.6)	70 (17.6)	68 (17.8)	2 (13.3)
MDS-UPDRS score				
Total (I + II + III)	36.0 ± 16.73	32.3 ± 12.91	32.6 ± 12.91	24.9 ± 8.87
Part I	5.4 ± 4.89	4.4 ± 3.61	4.4 ± 3.64	5.1 ± 2.81
Part II	6.2 ± 4.93	5.3 ± 3.92	5.4 ± 3.95	3.7 ± 2.55
Part III	24.4 ± 10.78	22.5 ± 9.12	22.8 ± 9.15	16.2 ± 5.53
Max UPDRS resting tremor	1.1 ± 0.96	1.2 ± 0.99	1.2 ± 0.99	0.9 ± 0.88
Posture stability score (UPDRS 3.12)	0.3 ± 0.72	0.1 ± 0.36	0.1 ± 0.37	0.0 ± 0.00
Hoehn and Yahr scale				
1	18 (24.0)	104 (26.3)	96 (25.2)	8 (53.3)
1.5	5 (6.7)	23 (5.8)	22 (5.8)	1 (6.7)
2	38 (50.7)	250 (63.1)	244 (64.0)	6 (40.0)
2.5	10 (13.3)	17 (4.3)	17 (4.5)	0 (0)
3	4 (5.3)	2 (0.5)	2 (0.5)	0 (0)
S&E ADL score	92.7 ± 6.81	92.2 ± 7.24	92.2 ± 7.30	92.7 ± 5.94
MoCA score	25.6 ± 3.96	27.4 ± 1.87	27.4 ± 1.88	27.4 ± 1.88
PASE score	Not available	168.3 ± 86.74	168.3 ± 86.74	Not available

Data are *N* (%) or mean ± SD

*DaT-SPECT* dopamine transporter single-photon emission computed tomography, *MDS-UPDRS* Movement Disorder Society revision of the Unified Parkinson’s Disease Rating Scale, *MoCA* Montreal Cognitive Assessment, *PASE* Physical Activity Scale of the Elderly, *PD* Parkinson’s disease, *PIGD* postural instability/gait difficulty, *S&E ADL* Schwab and England Activities of Daily Living, *TD* tremor dominant

^a^ Termination of PD medication use must have occurred at least 12 weeks prior to day 1 of the study and for a maximum total duration not exceeding 30 days

### DaT-SPECT findings

The mean ± standard deviation (SD) injected ^123^I-ioflupane dose was 4.77 ± 0.397 mCi and the mean ± SD time between injection and scan was 3 h:51 min ± 20 min. No severe adverse effects related to ^123^I-ioflupane were reported. A grade 1 mild adverse event was reported (vasovagal response) that resolved spontaneously.

Following visual read of DaT-SPECT images acquired from the 398 participants, 15 (3.8%) were deemed to have a “normal” DaT-SPECT and 383 (96.2%) participants were deemed to have an “abnormal” DaT-SPECT (see Table 2 for classification). Seven scans required a consensus read and all seven were ultimately deemed to be “abnormal”). Participants with “normal” reads came from 12 different sites across seven countries. The demographics and clinical scores for the “normal” and “abnormal” DaT-SPECT cohorts are shown in Table 1. In general, demographics between the two groups were similar. Of the metrics compared, the “normal” DaT-SPECT group had statistically significant lower Movement Disorder Society UPDRS scores (summed I + II + III and the Part III subscale, *p* < 0.05, Wilcoxon rank sum test), and lower prior use of PD medication (*p* < 0.05, Fisher’s exact test).

**Table 2:** DaT-SPECT visual read results of the imaged participants (*N* = 398)

Classification	Activity pattern	Participants, *N* (%)
Normal	Activity in the putamen and caudate nuclei of both hemispheres is still visible	15 (3.8%)
Abnormal	Activity in the region of the putamen is absent or greatly reduced in at least one hemisphere. Activity is still visible in the caudate nuclei of both hemispheres	81 (20.3%)
	Activity is absent in the putamen of both hemispheres and confined to the caudate nuclei	204 (51.3%)
	Activity is absent in the putamen of both hemispheres and greatly reduced in one or both caudate nuclei	98 (24.6%)

*DaT-SPECT* dopamine transporter single-photon emission computed tomography

### DaT-SPECT quantification

**Fig. 1:**
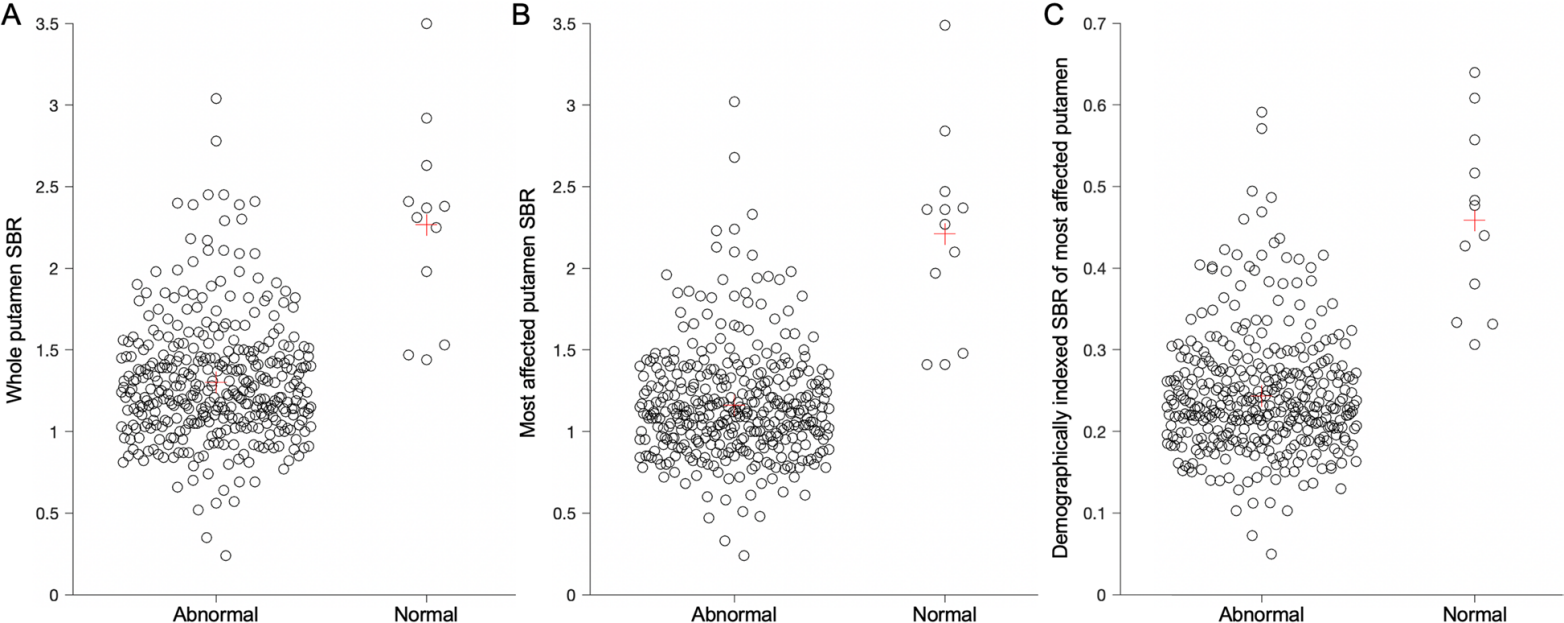
Quantitative evaluation of DaT-SPECT striatal binding ratio (SBR). **a**Whole putamen SBR, **b** most affected putamen SBR, and **c** demographically indexed most affected putamen SBR of participants in SPARK categorised by qualitative visual assessment as “abnormal” or “normal.” Demographic indexing normalises metrics to published normal SBR values based on age and sex [42]. Red cross indicates the mean value

The distribution of whole putamen SBR values for the two cohorts of participants is shown in Fig. 1. The mean putamen SBR was 1.30 ± 0.37 and 2.27 ± 0.61 in the “abnormal” and “normal” participants, respectively. This difference in means was statistically significant (two-tailed t-test, *p* < 1 × 10^−9^). Other regional means and asymmetry metrics derived from the quantitative image analysis are shown in Table 3.

**Table 3:** DaT-SPECT quantitative metrics

	DaT-SPECT screened	DaT-SPECT “abnormal”	DaT-SPECT “normal”
Quantifiable, *N*	389	377	12
Striatal binding ratio			
Whole striatum	1.37 ± 0.37	1.35 ± 0.35	2.03 ± 0.54
Whole putamen	1.33 ± 0.41	1.30 ± 0.37	2.27 ± 0.61
Whole caudate	1.40 ± 0.37	1.39 ± 0.36	1.79 ± 0.49
Most affected putamen	1.19 ± 0.39	1.16 ± 0.33	2.21 ± 0.61
Demographically indexed most affected putamen	0.25 ± 0.08	0.24 ± 0.07	0.46 ± 0.11
Abnormal whole striatum uptake, %	99.4	100	83
Abnormal whole putamen uptake, %	99.4	98.7	75
Abnormal whole caudate uptake, %	100	100	100
Striatal asymmetry index	4.30 ± 2.85	4.40 ± 2.84	1.17 ± 0.70
Abnormal striatal asymmetry index, %	0	0.5	0

Data are mean ± SD unless otherwise noted

*DaT-SPECT* dopamine transporter single-photon emission computed tomography

## Discussion

The present study deployed DaT-SPECT as a PD enrichment biomarker and revealed a SWEDD incident rate (3.8%) that was significantly lower than rates typically observed in several large multicenter studies with similar PD populations [6–8, 26, 44, 45]. Previous studies had observed a near-linear inverse relationship between SWEDD rate and disease duration [25]. However, the observed SWEDD incidence rate for the SPARK population, with an average disease duration of 8.4 months, did not follow this anticipated relationship. It is important to note that the lower incidence of SWEDDs in this trial occurred despite the fact that the SPARK diagnostic criteria for PD allowed for the potential enrollment of participants with ostensibly early-stage disease.

The interpretation of the SWEDD incidence observed in the present study should be considered in the context of methodological differences between the present and previously published studies. First, SPARK was a large, multisite, global study conducted with standardisation of participant recruitment, image acquisition, processing, and read qualification. As such, the SWEDD rate was less impacted by possible variance in dose, image parameters, or variance owing to local practice standards compared to prior studies with smaller sample sizes. Second, the study population may be different from those studied previously. Large-scale clinical trials that test disease-modifying PD therapies, such as this study, are vulnerable to selection bias in that physicians may be more confident in selection of study participants, and symptomatic individuals with more probable PD diagnosis may be more motivated to participate. SPARK investigators may have had involvement in previous trials or experience in their own practice that could allow them to recruit more efficiently in this study – with the possibility of participants already being known to the sites before screening versus coming from the community via advertising. Furthermore, as DaT-SPECT is widely used diagnostically in clinical practice, it is not known how many participants had already had DaT-SPECT prior to enrollment, for whom the study imaging was merely confirmatory. These factors likely limited the numbers of non-PD participants from reaching the DaT-SPECT screening stage, resulting in a relatively lower SWEDD incidence rate.

Methodological differences related to DaT-SPECT radioligand and analytic approaches can also be considered when comparing SWEDD rates between available study reports. Although there is no indication that SWEDD determination is tracer dependent, the impact of different tracers across studies cannot be excluded from consideration. Many studies, including the PPMI and this study deployed the ^123^I-ioflupane ligand. However, some prior studies identified SWEDD participants by utilising ^123^I-ß-CIT or ^18^F-Dopa radioligands [6, 8, 26]. Regarding differences in image analysis/interpretation, several previous studies used quantitative or semiquantitative methods to classify participants with objective criteria [10, 33]. Because visual reads remain the standard for DaT assessment in clinical practice and trial settings [27], qualitative assessment was selected for determination of eligibility in the present study. Note though, that semiquantitative methods in some studies have been associated with relatively low rates of SWEDD classification [33]. This was also the case when applying a fully automated approach to the present data. While the mean striatal SBR significantly differed between the DaT-SPECT cohorts, when applying an age correction factor, 83% of the “normal” participants would have been classified as “abnormal.”

Though it is difficult to identify which factor or combination of factors resulted in the relatively low SWEDD incidence observed in this study, the result supports the view that experienced movement disorder specialists serving as principal investigators in this clinical trial were particularly adept in identifying individuals with early-stage PD via clinical assessment alone [31–33]. DaT-SPECT has utility in the differentiation of PD from other nondegenerative parkinsonian disorders (e.g., essential tremor); however, it has more limited value in differentiating among degenerative causes of other parkinsonian syndromes (e.g., multiple system atrophy, progressive supranuclear palsy, and dementia with Lewy bodies) [35]. Therefore, at present and without additional long-term studies, clinical assessment remains the most important tool in evaluating and diagnosing participants. If DaT-SPECT had not been considered as part of the SPARK inclusion/exclusion criteria, approximately 4% of the imaged participants would not have been excluded from the trial. This rate of exclusion is low in comparison with exclusion rates derived from enrichment markers deployed in neurodegenerative trials of other diseases. For example, ~ 38% of participants were excluded on the basis of amyloid PET imaging (“amyloid negative”) in a previous phase 1 Alzheimer’s disease trial [46]. Thus, when considering the radiation risks, imaging costs, and operational burden, the added value of DaT-SPECT as an enrichment marker in the present trial was relatively small.

It is important to consider that this report only includes baseline imaging results. Repeat imaging would be needed to confirm that the low rate of abnormal imaging predicts a corresponding low rate of cases with no progression of DaT deficit over time. In light of these and the aforementioned limitations, our results are not sufficient in themselves to support a reconsideration of the current approach to clinical trial enrollment. Moreover, the longitudinal evaluation of nigrostriatal degeneration via DaT-SPECT to assess drug efficacy may prove to be of value in assessing efficacy of treatments aiming at slowing PD progression. Further, DaT-SPECT may confer value for potential prodromal PD trials aiming to recruit individuals with normal striatal binding, and subsequently using a prospective change in DaT-SPECT classification as an outcome measure. While DaT-SPECT has potential value as a research tool, the current literature, particularly in light of our results, does not support the need for DaT-SPECT prior to treatment with an approved drug in clinical practice. A requirement for DaT-SPECT confirmation prior to clinical treatment in routine practice would add unnecessary expense in the vast majority of cases and limit access for patients who could potentially benefit from a hypothetical approved therapy.

## Conclusion

To conclude, a low SWEDD incidence rate was observed in this trial. It is possible that study design aspects (e.g., clinical, logistical, and oversight) may have enriched participant selection. Further research is needed to understand the reasons for the low SWEDD rate in this study and whether these results are generalisable to future studies. DaT-SPECT remains the most accepted tool for PD trial enrichment. However, upon consideration of the SWEDD rate findings observed in the SPARK trial, we suggest that decisions regarding the value and cost-effectiveness of DaT-SPECT as an enrichment biomarker in future PD trials be weighed carefully with other factors that may serve to enrich the study population related to site expertise, nature of the participant recruitment pool, disease stage, and level of trial oversight.

## Data Availability

Data are available on request at http://clinicalresearch.biogen.com.

## Abbreviations

DaT: Dopamine transporter
PET: Positron emission tomography
PD: Parkinson’s Disease
SPECT: Single-photon emission computed
SBR: Striatal binding ratio
SWEDD: Scans without evidence of dopaminergic deficit
